# Who pays for health care in Ghana?

**DOI:** 10.1186/1475-9276-10-26

**Published:** 2011-06-27

**Authors:** James Akazili, John Gyapong, Diane McIntyre

**Affiliations:** 1 Navrongo Health Research Centre, Ghana Health Service, P. O. Box 114, Navrongo, Ghana; 2 Research and Development Division, Ghana Health Service, Accra, Ghana; 3 Health Economics Unit, Department of Public Health and Family Medicine, Faculty of Health Sciences, University of Cape Town, South Africa

## Abstract

**Background:**

Financial protection against the cost of unforeseen ill health has become a global concern as expressed in the 2005 World Health Assembly *resolution *(WHA58.33), which urges its member states to "plan the transition to universal coverage of their citizens". An important element of financial risk protection is to distribute health care financing fairly in relation to ability to pay. The distribution of health care financing burden across socio-economic groups has been estimated for European countries, the USA and Asia. Until recently there was no such analysis in Africa and this paper seeks to contribute to filling this gap. It presents the first comprehensive analysis of the distribution of health care financing in relation to ability to pay in Ghana.

**Methods:**

Secondary data from the Ghana Living Standard Survey (GLSS) 2005/2006 were used. This was triangulated with data from the Ministry of Finance and other relevant sources, and further complemented with primary household data collected in six districts. We implored standard methodologies (including Kakwani index and test for dominance) for assessing progressivity in health care financing in this paper.

**Results:**

Ghana's health care financing system is generally progressive. The progressivity of health financing is driven largely by the overall progressivity of taxes, which account for close to 50% of health care funding. The national health insurance (NHI) levy (part of VAT) is mildly progressive and formal sector NHI payroll deductions are also progressive. However, informal sector NHI contributions were found to be regressive. Out-of-pocket payments, which account for 45% of funding, are regressive form of health payment to households.

**Conclusion:**

For Ghana to attain adequate financial risk protection and ultimately achieve universal coverage, it needs to extend pre-payment cover to all in the informal sector, possibly through funding their contributions entirely from tax, and address other issues affecting the expansion of the National Health Insurance. Furthermore, the pre-payment funding pool for health care needs to grow so budgetary allocation to the health sector can be enhanced.

## Background

Health care financing strategies have recently been given greater priority in international health policy debates and research [1]. A consensus is emerging on the need for developing countries to move towards universal coverage through pre-payment financing mechanisms, given that user fees and other direct payments have had and continue to have negative effects, particularly on poor individuals and households [2,3]. User fees and direct payments disproportionately affect the poor. Unfortunately exemptions that were introduced to try to cushion the effects of user fees have failed to protect the poor from catastrophic health care costs to the point that 84% of those eligible for exemptions in Ghana never got them [4]. Evidence also shows that simply removing user fees, as some advocate, is not a sustainable solution to health care financing. It has to be supported by a simultaneous increase in funding through pre-payment mechanisms [5]. There is therefore a growing need for developing countries, particularly in Africa, to ensure fair financing in their health systems, and provide universal coverage with financial protection for their populations if they are to achieve the health-related MDG goals by 2015 (which is less than five years away). WHO has recognized this need and in its World Health Assembly resolution WHA58.33 called on all member states to "plan the transition to universal coverage of their citizens" [6]. Identifying a combination of health care financing mechanisms that would provide the needed access to health care services for all citizens is best informed by understanding how the burden of health care financing currently falls on different segments of the population.

Although there is a commitment to pursuing a universal health system in Ghana, no assessment of equity in health care financing has been undertaken. To improve equity in health care financing and promote the goal of achieving universal coverage, there is a need to measure the degree of progressivity of *existing *health care financing mechanisms to be able to establish the relative funding burden on the poor compared with the rich. This will allow us to identify which health care financing strategies are regressive (i.e. place a greater burden on the poor) and which are progressive (i.e. the rich contribute a higher proportion of their income than the poor). It will therefore provide insights into which financing mechanisms best provide financial protection and promote universal coverage.

The paper thus seeks to investigate the extent to which paying for health care relates to people's ability to pay and to investigate the relative progressivity of each of the financing mechanisms. The paper also assesses the overall progressivity in Ghana's health financing system. The application of relevant tools for measuring the equity of financing mechanisms, particularly for assessing the progressivity of financing mechanisms, has remained focused primarily on the health care systems of developed countries and, more recently, some Asian countries [7,8]. There has been only very limited application in developing countries and almost none in sub-Saharan Africa [9,10]. This is despite the apparent importance of health care financing equity as a central policy goal in many health systems in developing countries. With the exception of Tanzania and South Africa, no such research has been carried out in Africa and so the study not only serves as the first comprehensive assessment of the relative progressivity of the various health financing mechanisms in Ghana but has broader relevance to other African countries.

## Data and Methods

The secondary data used for the financing incidence calculations were the latest Ghana Living Standards Survey (GLSS) of 2005/2006 collected by the Ghana Statistical Service, a national body responsible for conducting all demographic, health and living standards surveys. This is the fifth time this survey has been conducted, hence its name of GLSS 5. The sample size for GLSS 5 was 8,687 households (see Table 1 for details). Data collected by a GLSS relates to all aspects of household decision-making and well-being. The nationally representative data contain information on household consumption of both durable and non-durable items. Data on the consumption of durable items were collected for the previous 12 months whilst those for non-durable or frequently purchased items were collected weekly for ten weeks using a weekly diary.

**Table 1:** Data source

	**Secondary**	**Primary**
Name of Survey	**GLSS**	**SHIELD survey**
Year of Survey	**2005/2006**	**2008**
Number of households	**8,687**	**2,986**
Number of individuals	**36,488**	**14050**
Sample as a % of total pop of Ghana	**0.17**	**0.007**
Weighted to national population	**Yes**	**Yes**
**Organisation**	**Ghana Statistical Services**	**Own field work**

To complement the GLSS data, a primary household survey^1 ^was conducted in a sample of six districts focusing on contributions to the NHI and direct health care payments. The SHIELD survey collected data on household expenditure on health care and household socio-economic status (SES) among other variables. The SHIELD household survey was weighted to be more representative of the national population, based on the proportion of rural and urban population as well as the insured and uninsured population in the three broad ecological zones of the country.

Analysis of financing incidence requires two key variables, the ability to pay or socio-economic status and the amount paid towards health care through various payment mechanisms. Each of them is examined in relation to how they were analyzed in this study.

### The ability to pay or socio-economic status (SES) variable

The study related health care payments to SES within households to determine the incidence of health care payments (i.e. health care payments as a percentage of household resources). The key issue is how best to measure SES or living standards. The approaches available for measuring SES that are applicable to the evaluation of the incidence of health care payments are household income and household consumption expenditure.

According to the literature, the most 'relevant' measure of SES must depend largely on the availability of the required data. As noted in a study in Nepal, there is no perfect measure of SES [11]. Income and consumption expenditure measures have each been used in different studies [8]. Reported income is often seen as a good measure of SES. The use of income as a measure of socio-economic status for example, allows one to examine income elasticity in health care payments. Income also measures the degree of household control over resources (which they could use if they so wish) [12].

However, income data have their drawbacks. First, the lack of an organized labour market particularly in sub-Saharan Africa and income variability over time does not allow it to be a good estimate of SES especially in developing countries like Ghana. Income could also be underestimated in developing countries with a large informal sector population and subsistence agriculture activities. Also for fear of taxes or other related issues, people tend to under report their income. Due to the drawbacks with income measurement, household consumption expenditure is often preferred in this kind of analysis. It is important to note that consumption expenditure also has its drawbacks. For instance households tend to under-declare what they spend on certain additive goods (e.g. alcohol, cigarettes) or illicit items (e.g. drugs, prostitution). Despite the drawbacks, consumption expenditure is a better measure than income particularly in developing countries with a large informal sector. In the first place, consumption expenditure smoothes out income irregularities and so reflects long-term average well-being. Secondly, consumption expenditure tends to be less understated than income because it is easier to provide expenditure information than income [13].

The construction of the socio-economic measure in this study is based on household's reported expenditure and consumption of food, housing and other non-food items. The measure also takes into consideration consumption from sources other than purchases from the market (e.g. subsistence agriculture products). The unit of analysis in the comprehensive health care financing incidence analysis and cross sectional case studies is the household. Households were divided into five quintiles for aspects of the analysis.

### Financing mechanisms-calculations

### Tax (direct and indirect) incidence analysis

An analysis of tax incidence is required in this study because the Ghanaian health care system, as in many other developing countries, is significantly financed by direct and indirect tax revenue.

Certain assumptions^2 ^have to be made about the tax-shifting element. These shifting assumptions facilitate the allocation of the burden of each tax payment to different income groups [14]. In this study, we assume that the incidence of direct tax (mainly personal income tax in Ghana) falls on the legal tax payer and indirect taxes (import, fuel levy, VAT) fall on the consumer. The only tax that has little agreement in the literature about its incidence assumption is corporate tax (CT) and due to the lack of consensus as to how corporate tax incidence should be calculated, a number of approaches have been put forward. Different authors have assumed the burden or incidence of corporate tax differently [13,14]. The key elements of the debate regarding this tax have been whether increases in corporate tax will result in lower wages, lower retail earnings or higher prices? Some writers [13-15] assume an equal share (50%) of the burden for consumers and shareholders (mainly foreign owned in Ghana) and this is applied in this study. The assumption of an equal share (50%) of the burden of corporate tax to consumers and shareholders is applied here because of the lack of consensus in the literature as to who ultimately bears the burden of corporate tax. The taxes identified and measured in this study included direct taxes (income tax and corporate tax), indirect taxes (VAT, National Health Insurance Levy (NHIL), fuel levy, import duty). These taxes make up over 95% of the total tax revenue collected in Ghana. In calculating the incidence of tax payments, each tax payment per household was estimated from relevant sections of the GLSS and triangulated with actual revenue from this tax as reported by the tax collector or Ministry of Finance.

### Non-tax health care financing incidence analysis

Apart from tax, health care in Ghana is also financed by health insurance contributions made up of premiums (through District Health Insurance Schemes-DHIS) from the informal sector and payroll deductions^3 ^(by Social Security and National Insurance Trust-SSNIT) to the National health insurance scheme as well as out-of-pocket (OOP) payments. Before allocating the above health care payments to income groups by quintiles of households, it is important to state the incidence assumption as to who bears the burden of each of the health care payments. The incidence of SSNIT contributions for instance falls on formal sector workers and that of the DHIS contributions fall on those who are in the informal sector who are insured. OOP payments are assumed to directly affect the consumer of the service. Most previous estimates of the incidence of OOP payments in developing countries have relied on data from small-scale health surveys that are not nationally representative and often restricted to rural areas [16,17]. We analysed data from the GLSS which has comprehensive information on health care and household consumption expenditure and which allow us to estimate the magnitude of the incidence of OOP payments in Ghana.

With regard to the incidence of the national health insurance (NHI) contributions, comprehensive data on health insurance contributions including premium and registration payments largely by the informal sector was collected through the SHIELD household survey in six districts in 2008. As the NHI was only introduced in 2004, the GLSS (conducted in 2005) did not capture much information on these payments, hence the need for the SHIELD survey. The premium contributions paid by the informal sector directly to the District health insurance schemes is graduated by law (Act 650). It ranges from ¢72,000 to ¢480,000 (US$8.00 to US$53.00) [18,19]. The graduation was designed in such a way that the poor should pay the lowest rate and the higher rates should be paid by the rich. However, in reality it appears that the premium payment generally is flat, due to the difficulty of assessing an informal sector household's ability-to-pay, which makes it all the more important to document the distribution of the burden of this payment mechanism across the population as a whole. The GLSS was used to predict the consumption expenditure variable in the SHIELD data. The assumption was made that the variable on frequent spending in the SHIELD data set was analogous to the frequent spending variables in the GLSS data. The expenditures on frequently purchased items, age of the head of the household and location (rural/urban and region) of the household were identified and used to predict the total consumption expenditure through a log regression model. The correlation of the prediction was about 80%. The prediction was based on the GLSS and coefficients obtained from this regression were used to predict the consumption expenditure in the SHIELD survey [20]. Table 2 provides a summary of each type of financing mechanism and the quantification technique. It should be noted that the 4.3% that constitute other taxes in Table 2 was allocated to the identified taxes according to their percentage share of the total taxes in the analysis. This technique was used in a similar study in South Africa [10].

**Table 2:** Financing incidence analysis estimation techniques

Component	Share in Total Health care financing	Source of Data	Rates^a^	Computation technique
**Taxes**	**47%**			
Personal Income Tax^c^	5.2%	GLSS, 2005/2006	5%-20% depending on income level.	Apply the appropriate tax rate and tax thresholds on the gross taxable income (salaries and wages received, income from business or professional practice/activities, part of dividends and interest received and/or accrued on deposits) of working age individuals within each household within the taxable range
Corporate Income Tax	7.1%	GLSS, 2005/2006	Rate is 28% and this is paid quarterly in the case of large companies.	Apportioning the total corporate tax receipts based on the Ministry of Finance data to households based on the tax shifting assumptions. Assumption of tax shifting includes certain percentage borne by shareholders (the GLSS collected information on those who receive dividends) and the rest by households through consumption. The tax shifting assumption was equal (50:50) tax burden shared between consumers and shareholders/capital owners
Value Added Tax (VAT)	11.3%	GLSS, 2005/2006	15% on standard rate goods and services	The VAT rate is applied to expenditure of goods and services that are standard rated excluding the zero-rated and exempted goods (since 2.5% is specially earmarked fund for education and 2.5% for health services, 10% was considered in the calculations)
National Health Insurance Levy	2.4%	GLSS, 2005/2006	2.5% on standard rated goods and services	The same distribution across households as VAT but at 2.5%. NHIL rate is applied to expenditure of goods and services that are standard rated excluding the zero-rated and exempted goods (the same goods and services as VAT).
Import Duty	8.0%	GLSS, 2005/2006	Varied depending on item	Comprehensive list of items subject ot import duty and amount received in duty for each item was obtained from Custom Excise and Preventive Service (CEPS) and this amounts were allocated to households based on reported consumption of these imported items from GLSS
Fuel Levy	8.5%	GLSS, 2005/2006	¢716.72/litre for petrol ¢429.96/litre for Diesel ¢353.88/litre for kerosene	Since fuel is consumed by households (for both personal and public transport) as well as corporate users, estimation involved a process of generating the component attributable to public transport users, users of private transport and those attributable to users in businesses
Other	4.3%	GLSS, 2005/2006	Includes taxes on cigarette, drinks, stamps, airport departures, and unidentified levies.	Not calculated (small share of total revenue)
**Insurance **	**5%**			
National Health Insurance Scheme	5%	SHIELD household survey 2008^b^		Total national health insurance contributions is made up of premium contributions of the informal sector and payroll deduction of formal sector workers
**Out-of-pocket payment**	**48%**			
OOP payments	48%	GLSS, 2005/2006		Comprehensive household expenditure on medicines, consultations, preventive and curative treatments, procedures excluding transportation were summed up

^a^This applies to the taxes and are based on the 2005/2006 assessment year: NB: GLSS-Ghana Living Standard Survey

^b^This was our own survey that was collected to complement the GLSS data and to answer key questions on the NHIS

^c^Rebates is not a common practice in Ghana *Inter-bank exchange rate = ¢9,072.12 to US$1.00 in 2005

### The Kakwani index for measuring progressivity of health care financing

In addition to estimating contributions to each financing mechanism as a percentage of consumption expenditure in each quintile, it was necessary to calculate the incidence of financing using the Lorenz and concentration curves to establish whether a health care financing mechanism is progressive, regressive or proportional relative to ability to pay (ATP) or SES. The Lorenz curve is a graphical representation of the cumulative distribution function of the empirical probability distribution of wealth or ATP or SES. The concentration curve plots the cumulative distribution of health care payments, while the concentration index is twice the area between the concentration curve and line of equality (the 45º line running from the bottom-left corner to the top-right). To enable the illustration of the degree of relative progressivity of each health care payment mechanism and the overall health financing incidence, we use the Kakwani index. Other methods like the Suits index could be used but the Kakwani index is more popular and widely used in this type of analysis [8,21]. The Kakwani index is defined as twice the area between the Lorenz curve for gross consumption expenditure (ATP or SES) and the concentration curve for health care payments [9,22]. Kakwani index was computed as the difference between the concentration coefficients of health care payments and the Gini coefficients of income (i.e. expenditure). The value of the Kakwani index ranges from -2 to 1 [22]. A positive Kakwani index indicates the health care financing system is progressive, so that the Lorenz curve lies above the concentration curve, and vice versa if it is regressive. A Kakwani index of zero indicates proportionality of health care payments and thus the Lorenz and concentration payments curves would coincide. According to Wagstaff and others, when the concentration curve for health care payments lies completely outside the Lorenz curve of ATP or SES (which in this case is based on household consumption expenditure), the health care payment is progressive. The opposite is true if it is regressive. Proportionality is attained when the two curves coincide [21]. Test of dominance, using the standard errors and point estimators of the concentration and Lorenz curves were performed to assess whether the difference between the concentration and the Lorenz curve or the 45 degree line is statistically significant [23].

## Results

### General taxes

### Direct taxes

**Figure 1:**
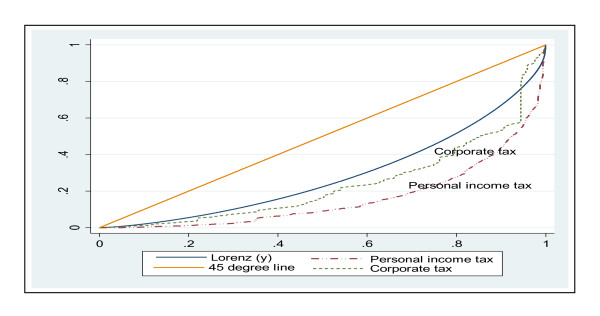
**Concentration curves of PIT and CT payments and Lorenz curve of household expenditure, Ghana: 2005/2006**.

Direct taxes are those paid directly to the revenue authorities. The main direct taxes in Ghana are Personal Income Tax (PIT) and Corporate Tax (CT). The concentration curve for PIT and CT (using the assumption that 50% of CT is distributed across household according to their reported consumption expenditure on manufactured goods and 50% across shareholders based on receipts of dividends) were constructed as well as the Lorenz curve for household consumption expenditure. As can be seen in Figure 1, the concentration curves of PIT and CT lie outside the Lorenz curve. In other words the Lorenz curve dominates the concentration curves of PIT and CT payments indicating that they are progressive. The Kakwani index of PIT and CT was calculated at 0.256 and 0.099 respectively (see Table 3) and this confirms the progressivity of PIT and CT.

**Table 3:** Cumulative shares of health payments by consumption expenditure quintile, Ghana (GLSS 2005/2006 & SHIELD 2008)

Per capita Household (HH) Expenditure Quintile	Per capita HH Expend	GLSS 2005/2006 Data										SHIELD data 2008
			
		Direct taxes (A)			Indirect taxes (B)					(C) Out-of-pocket Payment	Total financing of A, B and C	
					
		Personal Inc tax	Corp Inc tax∞	Direct tax	VAT±	Import duty	Fuel levy	Kerosene levy	Indirect tax			NHI contributions
Poorest 20% (Standard error)	**5.53%** (0.096)	1.25%* (0.208)	3.38%* (0.807)	1.99%* (0.271)	3.26%* (0.931)	4.02%* (0.496)	6.43%* (0.197)	16.76%* (0.376)	4.07%* (0.570)	**6.92%** (1.810)	**4.33%** (-0.88)	2.91%* (0.371)

Poorest 40% (Standard error)	**15.64%** (0.2371)	6.36%* (1.616)	10.70%* (2.469)	7.85%* (1.294)	8.91%* (1.930)	11.48%* (1.411)	17.97%* (0.425)	37.82%* (0.594)	11.34%* (1.330)	**19.36%** (2.758)	**12.85%** **(-1.79)**	10.06%* (0.994)

Poorest 60% (Standard error)	**30.38%** (0.4288)	13.01%* (1.865)	**23.58%** (4.962)	16.66%* (1.910)	19.42%* (4.399)	22.04%* (2.691)	33.88%* (0.676)	60.49%* (0.685)	22.85%* (2.758)	**37.72%** (4.647)	**25.74%** **(-3.11)**	19.31%* (1.344)

Poorest 80% (Standard error)	**51.64%** (0.6813)	27.84%* (2.651)	**44.05%** (8.462)	33.43%* (3.131)	**53.54%** (9.624)	39.78%* (4.851)	54.60%* (0.960)	81.80%* (0.630)	**49.47%** (5.574)	**57.98%** (6.395)	**46.96%** **(-5.03)**	38.42%* (2.134)

Test of Dominance-Against 45% line	-	-	-	-	-	-	-	-	-	-	-	-

-Against Lorenz C		-		-	-		+	+	-		-	-

Concentration index/Gini coeff	0.424	0.680	0.522	0.625	0.473	0.552	0.383	0.016	0.481	0.354	0.487	0.567

(Robust SE)	(0.019)	(0.040)	(0.087)	(0.037)	(0.074)	(0.038)	(0.014)	(0.013)	(0.041)	(0.059)	(0.046)	(0.031)

*(p-value)*	(0.000)	(0.000)	(0.000)	(0.000)	(0.000)	(0.000)	(0.000)	(0.000)	(0.000)	(0.000)	(0.000)	(0.000)

Kakwani index		***0.256***	***0.098***	***0.202***	***0.049***	***0.129***	***-0.041***	***-0.408***	***0.057***	***-0.070***	***0.064***	***0.143***

(Robust SE)		(0.112)	(0.207)	(0.101)	(0.144)	(0.082)	(0.036)	(0.034)	(0.081)	(0.093)	(0.092)	(0.074)

*(P-value)*		(0.022)	(0.632)	(0.046)	(0.733)	(0.115)	(0.261)	(0.000)	(0.477)	(0.463)	(0.329)	(0.051)

Source: Author;

Note: For shares: **Bold **indicates significant difference from population share (5%)

* indicates significant difference from expenditure share (5%)

(Standard errors for concentration and Kakwani indexes are robust to heteroskedasticity and within cluster correlation)

∞ Assumption is that corporate tax is distributed equally (50/50) across households (based on reported consumption of manufactured goods) and shareholders (based on receipt of dividends) ** NHIL: National Health insurance levy (2.5% of social security of formal workers)

# Dominance test (- indicates the 45% degree line/Lorenz curve dominates the concentration curve: + indicates concentration curve dominates 45%-degreeline/Lorenz curve. Blank indicates non dominance. ± NHIL has same results as that of VAT

### Indirect taxes

**Figure 2:**
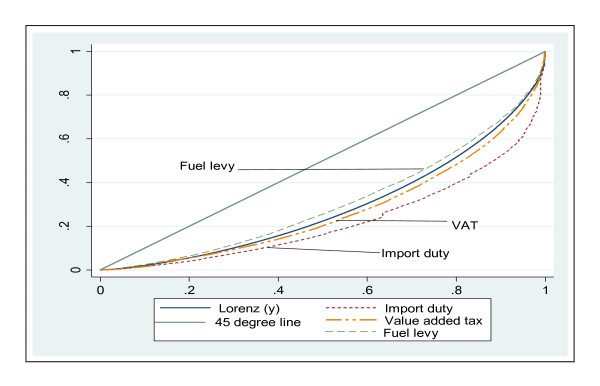
**Concentration curves of Fuel, VAT and Import duty payments and Lorenz curve of household expenditure, Ghana: 2005/2006**.

The main indirect taxes in Ghana in 2005/2006 were VAT (including the national health insurance levy (NHIL) which is a component of VAT), fuel levy and import duty. Figure 2 depicts the results of the indirect taxes and as can be seen, the concentration curve of VAT lies outside of the Lorenz curve of consumption expenditure. The Kakwani index (Table 3) is positive confirming progressivity. With regard to the fuel levy (composed of levies from petrol, diesel, engine oil, kerosene and other lubricants), the concentration curve dominates the Lorenz curve, suggesting that the fuel levy is regressive (see Figure 2). The Kakwani index for the fuel levy was -0.041 (see Table 3) and this means that the fuel levy in Ghana is regressive. In terms of import duty, the concentration curve is completely dominated by the Lorenz curve. To obtain the magnitude of the progressivity of import duty, the Kakwani index was calculated and has a value of 0.129.

### Total tax

**Figure 3:**
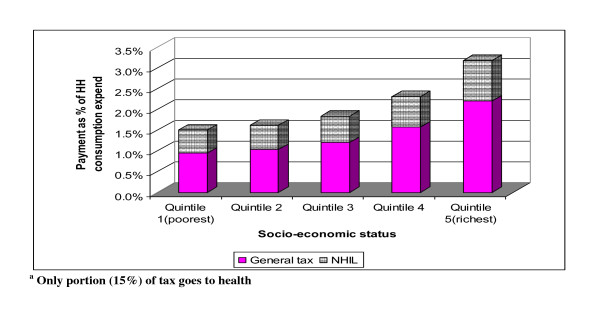
**General tax^a ^and NHIL payment as a proportion of household consumption expenditure by quintile (based on per capita)**.

Pooling all the components of general tax together (direct and indirect), but excluding the NHIL and education earmarked tax, the incidence of tax overall was calculated. Figure 3 shows that overall taxation is progressive. Figure 3 shows the incidence of that *portion *of general taxes that goes to health as well as the NHIL. It is to be noted that government allocated 15% of tax revenue to the health sector in 2005/2006. The only tax to which the 15% figure was not applied was the NHIL as this is an earmarked tax that goes in total to the health sector. The results shows that tax overall is progressive (Kakwani index = 0.130).

**Figure 4:**
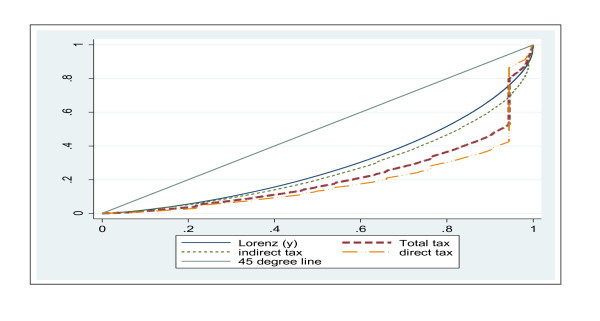
**Concentration curves of Total tax payments and Lorenz curve of household expenditure, Ghana: 2005/2006**.

The concentration curves for direct, indirect (excluding NHIL and dedicated education tax) and overall taxes are compared with the Lorenz curve of consumption expenditure (see Figure 4). Direct tax is the most progressive as is revealed by the magnitude of the dominance of the Lorenz curve over the concentration curve. The concentration curve for direct tax lies well outside the Lorenz curve (Figure 4).

### Non-tax health care payments

**Figure 5:**
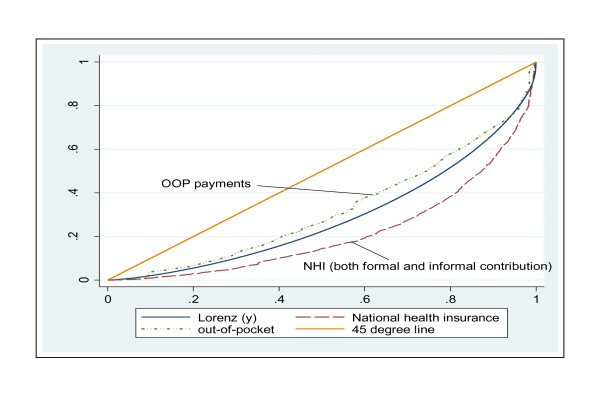
**Concentration curves of OOP and NHI payments and Lorenz curve of household expenditure, Ghana: 2005/2006**.

Apart from allocations from tax revenue, the Ghanaian health system is financed by direct out-of-pocket (OOP) payments (accounting for nearly half of all health care expenditure) and health insurance (composing of premiums and payroll deductions). The concentration curves for OOP and health insurance were constructed and as can be observed in Figure 5, OOP payments are a regressive means of funding health care in Ghana. This is because the concentration curve of OOP payments dominates the Lorenz curve throughout the distribution except for the last section (top right corner) where the two curves (Lorenz and concentration) appear to coincide (see Figure 5). The regressivity of OOP payments is confirmed by the negative value for the Kakwani index (-0.070). In contrast, contributions to the NHI are progressive with the burden of these payments rests more on the rich than the poor. The concentration curve of total NHI contributions confirms the progressivity of the total NHI contributions (Figure 5). The concentration curve lies completely outside the Lorenz curve. Further confirmation of the progressivity of the total NHI contribution is shown in Table 3, where the Kakwani index is positive (0.144). It is important to mention that NHI contribution from the formal sector is progressive (Kakwani index = 0.256) whilst the informal NHI contributions (premiums) are regressive (Kakwani index = -0.307).

### Total health care financing incidence

Table 3 combines all sources of health care payment except NHI contributions in Ghana (general tax, NHIL and out-of-pocket payments) and measures their total payments as a proportion of consumption expenditure. The table also provides summary statistics of the Gini coefficient of the consumption expenditure and the concentration index of health care payments from which the Kakwani indices are calculated.

Table 3 shows that health care financing is generally progressive (i.e. the combined effect of general tax, NHIL and out-of-pocket payments from GLSS 2005/2006 data and the NHI contributions from SHIELD 2008 data). It should be noted again that only a portion of total government tax resources (i.e. 15% in 2005/2006) was allocated to the health sector and this is factored into the calculation of the general tax component. The Kakwani index of 0.064 for the combined general tax, NHIL and out-of-payment and 0.143 for the NHI contributions shows that health care financing is Ghana is generally progressive. It can be seen from Table 4 that the poor (Quintile 1 and 2) are nevertheless making substantial contributions of their meagre income to health care, particularly through OOP payments.

**Table 4:** Distribution of total health financing as a proportion of household consumption expenditure by quintile in Ghana

	Financing sources			
	
Socio-economic status	General tax^a^	NHIL^a^	NHI contributions^b^	OOP payments^a^
Quintile 1 (poorest)	1.50%	0.55%	0.08%	2.87%
Quintile 2	1.62%	0.59%	0.09%	2.83%
Quintile 3	1.84%	0.64%	0.09%	2.61%
Quintile 4	2.31%	0.73%	0.11%	2.18%
Quintile 5 (richest)	3.17%	0.98%	0.13%	2.76%

^a^GLSS 2005/2006 data ^b^SHIELD 2008 data

## Discussion

African countries face many challenges in health care financing, not least being the inadequacy of funding and the high direct out-of-pocket payments for health care. In this context, there is a critical need for a comprehensive analysis of the progressivity of health care financing to inform debate on alternative health care financing approaches. This is especially the case for Ghana at a time when the country is still in the process of restructuring its health care financing in the wake of the introduction of a national health insurance system. The findings show that direct taxes, which comprise personal income tax and corporate tax, are progressive with positive Kakwani indices. The progressivity of direct tax is consistent with findings elsewhere (see Table 5).

**Table 5:** Cross-country comparison of progressivity indices (distributional incidence of health care financing)

Country	Year	Financing mechanisms				Total financing
			
		Direct tax	Indirect tax	NHI/SHI contributions	OOP or direct payments	
**Africa**						

**Ghana**	**2005/2006**	**0.202**	**0.057**	**0.144**	**-0.068**	**0.071**

South Africa	2005/2006	-	-	-	-0.0259	0.065

**Asian countries**						

Bangladesh	1999/2000	0.552	0.111	-	0.219	0.214

Thailand	2002	0.510	0.182	0.180	0.091	0.197

Phillippines	1999	0.381	0.002	0.205	0.139	0.163

Malaysia	1998/1999	0.395	-0.078	0.081	0.104	0.186

Taiwan	2000	0.244	0.040	-0.075	-0.079	-0.029

Sri-Lanka	1996/1997	0.569	-0.010	-	0.069	0.085

Indonesia	2001	0.196	0.074	0.306	0.176	0.173

China	2000	0.152	0.040	0.235	-0.017	0.040

Nepal	1995/1996	0.144	0.114	-	0.053	0.063

Japan	1998	0.100	-0.223	-0.042	-0.269	-0.069

**OECD countries**						

Portugal	1980	0.279	0.079	0.277	-0.158	0.063

The Netherlands	1987	0.185	-0.009	-0.002	-0.059	-0.034

Spain	1980	0.170	0.023	-0.063	0.016	-0.023

Italy	1987	0.054	0.001	0.028	-0.004	0.022

USA	1981	0.162	-0.174	-0.035	-0.387	-0.145

UK	1985	0.131	-0.059	0.043	-0.190	0.032

Ireland	1987	0.250	-0.120	0.110	-0.070	0.034

Source: (Ataguba, McIntyre 2009 [1]; Wagstaff, van Doorslaer et al. 1999 [26]; O'Donnell, van Doorslaer et al. 2008 [8]); Authors own calculations for Ghana

Indeed in almost all countries examined to date, personal income tax is progressive in essence because these taxes are explicitly structured to be progressive. Corporate tax in Ghana was found to be progressive, but has a lower Kakwani index than personal income tax. Taken together, this means of course that direct taxes are progressive which is consistent with the results of other published empirical studies [8,24,25].

In contrast, the international evidence (see Table 5) on indirect taxes suggests that these tend to be regressive in some countries but progressive in others. They have been found to be regressive in Sweden, Denmark, Japan, Sri Lanka and South Africa [8,10,26] but progressive in many low- and middle-income countries in Asia (e.g. Bangladesh, Thailand and China) [8]. In Ghana, indirect taxes analyzed in this study include import duty, fuel levy, VAT and the National Health Insurance Levy (NHIL). With the exception of the fuel levy, the other elements of indirect taxes were found to be progressive (see Table 3). Fuel is regressive because of the influence of kerosene taxes, which is largely consumed by the poor. VAT is progressive because of the wide range of exemptions on agricultural goods and other goods largely consumed by the poor and that many goods purchased in rural markets escape the VAT 'net'. The progressivity of VAT is important in Ghanaian health care financing since the NHIL, which is the main source of the NHIS is a component of VAT. However the Kakwani index is less than 0.10. It is necessary to sound a warning here that if, as is likely, the economy becomes more formalized and more people end up paying VAT, VAT and the NHIL might well move to being proportional or even regressive. This possible change needs to be acknowledged and there is a need for continual monitoring of the incidence of this financing mechanism (NHIL) so that Ghana does not end up using a regressive financing mechanism to fund the NHI. Considering all the indirect taxes together, indirect tax is progressive as demonstrated by a positive Kakwani index. This is consistent with other low-income countries like Bangladesh, Malaysia, Thailand and Tanzania [8,24,27].

The national health insurance contributions, which are made up of formal sector payroll deductions and informal sector premium contributions, are progressive overall. This progressivity is largely a function of the payroll deductions. However, the informal sector's premium contributions, which are the basis for the expansion of the NHI to universal coverage, were found to be very regressive. Thus, within the informal sector, the poor are bearing the brunt of the NHI contributions (relative to their available household resources) compared to their richer counterparts. This is because everybody in the informal sector pays the same contribution, which in turn is the result of the failure to implement the system of graduated premiums and full exemptions for the poorest that was part of the original design of the national health insurance scheme (NHIS). This finding means that policy makers, government and all stakeholders of the NHIS must review the design of the NHIS and its implementation, not just the financing per se but also the other institutional arrangements such as the graduation of premiums and the policy on the operation of exemptions, which are simply not functional at present. The NHIS financing arrangements are supposed to be pro-poor, but as the scheme operates currently, they are not [28].

Apart from taxes and the NHI, the Ghana health care system is largely funded by direct out-of-pocket (OOP) payments. These accounted for 48% of total health care financing in 2005/2006. This form of financing has been found (with few exceptions) to be regressive (see Table 5). Indeed as is the case in Ghana, OOP payments have been found elsewhere to be more regressive (or at best, less progressive) than any other form of health care financing [8,26]. Even in countries (such as Bangladesh, Indonesia, Philippines and Korea) where OOP payments are progressive [8], the reason for this is that poor households simply cannot afford to pay for health care and therefore do not access health services. In other words, the seemingly 'progressive' OOP payments can be simply attributed to the fact that the poorest of the poor do not use health services when they are required to pay at the point of service delivery.

On the whole, health care financing in Ghana was found to be progressive. This finding is largely driven by the progressivity of most forms of tax, which make up close to 50% of total health financing. It remains the case, however, as the paper has shown that some taxes such as the fuel levy and out-of-pocket payments are regressive thereby diminishing the level of overall progressivity in health care funding.

The results presented in the previous section thus provide the answer to the key question "who pays for health care in Ghana?" The brief answer is that it is largely the better-off who pay for health care financing; however, the poor are also making substantial contributions relative to their household resources towards health care financing in Ghana, as can be observed in table 3 and 4.

## Conclusion

Interest in the distribution of health care payments across socio-economic groups arises in part due to its potential redistributive effect, particularly in terms of compulsory contributions towards health financing (e.g. through tax and national health insurance). Progressive financing takes proportionately more from the rich than the poor and could leads to a more equal the post-tax distribution of income. The paper assessed the incidence of health care financing in Ghana employing concentration curves and Kakwani's progressivity indices. This analysis represents the first study in West Africa to measure the progressivity of each of the health care financing sources and of the whole health care financing system in a comprehensive manner.

In terms of an assessment of the equity of health care financing in Ghana, this study highlights the regressivity of out-of-pocket payments. It was the recognition by government of the heavy burden of these payments and the barrier they create to health service access that prompted the introduction of the national health insurance in Ghana. However, the fact that OOP payments still constitute almost half of total health care expenditure and the extent of their regressivity highlights the need for more concerted efforts to increase the share of pre-payment financing mechanisms. Although, exemptions and waivers were advocated and implemented to reduce the burden of OOP payments, their effects have largely been negative due to poor implementation (e.g. lack of clarity in policy strategy) and insufficient funds [2,4,29].

There are a number of positive messages from this study in terms of the NHI. Firstly, the NHI levy, which is part of VAT, is currently progressive, but this may change in future and requires ongoing monitoring. Second, the contributions by formal sector workers are also progressive. However, the major concern in relation to the NHI is that contributions made by the informal sector are highly regressive. These contributions constitute a very small share of total NHI revenue (about 5%) and the government is currently considering instituting a 'one-time payment' for those in the informal sector [20], which will effectively translate into tax funding of the contributions for all outside the formal sector. The finding of the regressivity of insurance contributions by the informal sector is also relevant to other low- and middle-income countries which are pursuing health insurance for the informal sector and/or are planning to implement mandatory health insurance on a contributory basis for the entire population as part of a strategy for moving to universal coverage.

For Ghana to attain adequate financial protection for its citizens and ultimately achieve universal coverage, it needs to extend pre-payment cover to all in the informal sector, possibly through funding their contributions entirely from tax and possibly increasing budgetary allocation to the health sector. While this is not a simple task, it is the road that must be travelled if Ghana is to achieve its goal of universal coverage.

## Competing interests

The authors declare that they have no competing interests.

## Authors' contributions

JA designed the study, performed the analysis and drafted the report; JG participated in the revision of the manuscript and DM supported the design and analysis of the study and undertook a critical review of the manuscript. All authors read and approved the final manuscript.

## Endnotes

1 This survey is call "Strategies for Health Insurance for Equity in Less Developed countries-SHIELD"(http://web.uct.ac.za/depts/heu/SHIELD/about/about.htm)

2 Conventional tax incidence studies compute tax incidence on the basis of annual data for income sources and expenditure patterns and also on the basis of several assumptions concerning how the different taxes are shifted to households either because they are consumers, producers or owners of factors of production (land, labour, and capital). These assumptions are known in the literature under different interchangeable names: "shifting assumptions" or "incidence assumptions," or "sources and uses side effects".

3 2.5% of formal workers' salaries go to the NHIF as their premium contributions to the national health insurance scheme (NHIS)
